# Multilevel model on longitudinal data analysis in determinants of CD4 cell count among antiretroviral therapy attendant of HIV infected adults follow up in Gondar Teaching Referral Hospital, Gonder, Ethiopia

**DOI:** 10.1186/s12981-020-00329-5

**Published:** 2021-01-15

**Authors:** Kindu Kebede

**Affiliations:** Department of Statistics, College of Computing and Informatics, Haramaya University, Harar, Ethiopia

**Keywords:** CD4 count, Acquired immunodeficiency syndrome, Antiretroviral therapy, Ethiopia, Multilevel model

## Abstract

**Background:**

Human immunodeficiency virus attacked an immune cell and the CD4 cell which is responsible for the body’s immune to infectious agents. Acquired immunodeficiency syndrome is one of the major public health problems in Sub-Saharan Africa including Ethiopia. The main objective of this study to identify the determinants of CD4 cell count among antiretroviral therapy attendants of infected adults follow up in Gonder teaching referral hospital, Gonder, Ethiopia implemented by SAS version 94.

**Methods:**

A retrospective cohort study was conducted on 216 regular follow up patients whose age greater than 14 years from December 1, 2012, to December 30, 2017. A multilevel model was used to identify the factors of CD4 cell count of patients and it considered variability between and within patients.

**Results:**

The mean with a standard deviation of weight, and a hemoglobin level of patients were 55.48 (10.21), and 18.25 (33.028) respectively. This study concluded that the variation for CD4 cell count existed between patients was 63% and the remaining 37% of variation existing within patients. In this study, the random coefficient time-varying covariate model was well fitted which shows weight and hemoglobin level were statistically significant predictors at a 5% level of significance for the log of CD4 cell count of patients.

**Conclusion:**

This study shows the hemoglobin level and weight of patients were statistically significant for the log of CD4 cell count of patients follow up in Gonder teaching referral hospital, Gonder, Ethiopia. Moreover, the result of the study shows that the log of CD4 count of patients increased when hemoglobin level and weight of patients increased. Hence, intervention should be given the ways to increase weight and hemoglobin levels of patients during follow up of antiretroviral therapy.

## Introduction

Acquired Immune Deficiency Syndrome (AIDS) is caused by HIV (Human Immune Deficiency Virus) which reduces a person’s ability to fight infection. HIV attacks an immune cell and the CD4 cell is responsible for the body’s immune response to infectious agents. An uninfected individual has around 1100 CD4 cells per milliliter of blood. These CD4 cells, decrease in number with time from HIV, so that an infected person’s CD4 cell count can be used to monitor the progression of the disease [1].

Women account for nearly half of the 40 million people living with HIV-1 worldwide which has a higher proportion existing in developing countries and the human immunodeficiency virus–type 1 (HIV-1) is epidemic for more than 20 years [2]. ART can be helpful for people who have AIDS when diagnosed and can be lifesaving. Treatment is important for people with HIV when people who start ART soon after they get HIV to experience more benefits from treatment than people who start treatment after they have developed AIDS [3].

Sub-Saharan Africa remains most severely affected, with nearly 1 in every 20 adults (4.9%) living with HIV and accounting for 69% of the people living with HIV in this worldwide. Although the regional prevalence of HIV infection is nearly 25 times higher in sub-Saharan Africa than in Asia, almost 5 million people are living with HIV in South, South-East and East Asia combined. After sub-Saharan Africa, the region most heavily affected the Caribbean and Eastern Europe and Central Asia, where 1.0% of adults were living with HIV in 2011 (UNAID 2012).

Immunological failure as CD4 count falls to the baseline or persistent CD4 levels of below 100 cells/mm^3^. Virologic failure as plasma viral load above 1000 copies/ml based on two consecutive viral load measurements after 3 months, with adherence support [4]. Globally, the annual number of people newly infected with HIV continues to decline, although this varies strongly between regions. In general HIV/AIDS is one of the major public health problems in Sub-Saharan Africa including Ethiopia, as one of these countries has been affected by the epidemic with a prevalence was 1.5% and 1.1% in 2011 and 2015 respectively that shows urban are more affected than rural areas while females are twice affected than male population with HIV [5]. The World Health Organization (WHO) defines clinical failure amongst adults and adolescents as new or recurrent clinical conditions indicating severe immunodeficiency (WHO clinical stage 4 conditions) after 6 months of effective treatment.

### Study design and methods

A retrospective cohort study design was used to collect relevant information’s from ART chart in order to address the objective of this study. Those HIV positive patients who are greater than 14 years old and start ART since December 1, 2012 and who has base line and at least three follow up period until December 30, 2017 included in this study.

### Study area and population

This study conducted at Gondar Teaching Referral Hospital in North-Western Ethiopia, Amhara Region. The population of this study included HIV positive adults who attend antiretroviral therapy at Gondar Teaching Referral Hospital.

### Data collection procedures

This study obtained data from a retrospective cohort study based on ART electronic data base and from the review of patient charts which contains socio-demography, laboratory and clinical information of all HIV patients under ART follow-up including a detailed antiretroviral therapy history from Gondar Teaching Referral Hospital among Antiretroviral Therapy (ART) follow up study. The target population was adults of HIV positive patients whose age greater than 14 years old who initiated on ART from December 1, 2012 to December 30, 2017 GC.

### Quality of data

The data quality was controlled by data collectors from ART section of the hospital. The controllers were taken intensive training by the Minister of Health for the different services.

### Variables include in the study

The longitudinal response variables for this study were CD4 cell count. Therefore, CD4 count has count variable nature due to this reason generalized linear mixed model with the link function log is performed. In addition the predictor variables were used in this study shows on Table 1.

**Table 1:** Predictor variables

Gender	0 = Female, 1 = male
Age in years	Continuous
Marital status	1 = single, 2 = married, 3 = divorced, 4 = widowed
Weight	Continuous
Adherence status	1 = good, 2 = fair, 3 = poor
WHO Clinic Stage	1 = Stage I, 2 = Stage II, 3 = Stage III, 4 = Stage IV
Functional status	1 = working, 2 = ambulatory, 3 = bedridden
Hemoglobin level	Continuous variable
TB screening	1 = positive, 2 = negative
Duration of ART	Count
Baseline CD4	Count

### Inclusion and exclusion criteria

Inclusion criteria:- Patients whose age was above 14 years old that are attending a minimum of three visit of HAART treatment in ART clinic for refilling their prescription and who were initiated on ART from December 1, 2012 to December 30, 2017 GC at Gondar Teaching Referral Hospital would be included in the study.

Exclusion criteria:- Patients whose age was below 14 years old that are attending HAART treatment in ART clinic for refilling their prescription, patients who are not registered in the ART clinic and who are not initiated on ART were not included in this study. In addition patients out of the study period are not included.

### Data collection methods and procedures

This study was used secondary data extracted from patient chart follow-up format and analyzed by SAS version 9.4 statistical software. But the chart prepared by national health organization.

### Missing data treatment

One of the biggest problems in longitudinal studies is missing data. However, multilevel analysis is no need to have complete dataset. Therefore, multilevel analysis is very flexible in handling missing data [6].

### Statistical analysis technique for longitudinal data

To assess the changes of outcome (s) over time to associated risk factors by using multilevel longitudinal data. But analyzing multilevel longitudinal data is complicated. For analyzing of this longitudinal data, both descriptive and inferential statistics were used. Multilevel analysis is (probably) the most robust and flexible of the three techniques [6]. Thus, in this study multilevel longitudinal methods were employed.

### Growth curve analysis

Growth curve analysis is offers a statistical framework for analyzing longitudinal data. Growth curve analyses are used to describe the patterns of change over time and to determine the number of visit time. More specifically, growth curve analysis in longitudinal data can estimate a best-fit line or curve to each individual’s responses over time [7].

There are few strict requirements for the types of data that might be analyzed using growth model. First, adequate at least 100 sample sizes are needed to reliably estimate then growth models are often preferred. Second, growth models typically require at least three repeated measures per individual. Third, for the typical method of estimation called maximum likelihood, it is assumed that the repeated measures are continuous and normally distributed.

### Uni-variate growth curve analysis

According [8], separate growth curves are constrained to have the same slope but allowed to have different intercepts over time. As a consequence, this method underestimates variability and overestimates test statistics when individuals have different slopes.

### Multilevel analysis

Longitudinal data are one example of a hierarchical structure; series of repeated measures over time at the lowest level is nested with the individual persons at the highest level. Such nested structures are typically strong hierarchies because there is much more variation between individuals in general than occasions within individuals [9].These repeated measures are taken at either fixed or varying occasion. The measurements taken as a fixed occasion, all individual provided measurements at the same set of occasions, usually regularly spaced, such as in our study every 3 months. When occasions are varying, we have different set of measures take at different points in time for different individuals [10].

### Uni-variate multilevel analysis

This analysis is used for exploring an individual’s variability on longitudinal data for responses in such random effects or multilevel modeling allows investigate two level of variability. Therefore, within and between subjects variability were analyzed for CD4count at each individual i = 1, 2… n [11]. These models were analyzed based on either of following mechanisms.

### Intercept only model

This is the simplest case of hierarchical model in which there are no explanatory variables at all. Then model has only an intercept term and variances at the measurement and individual level. Since the model does not contain a slope, the true individual change is a horizontal line with y-intercept β0i.

The model expressed as:-

1 $$\begin{array}{*{20}l} {{\text{Level}} - 1:{\text{ Yti}} = \pi_{0} {\text{i}} + {\text{e}}_{0} {\text{i}}\quad {\text{where}},{\text{ e}}_{\text{ti}} \sim {\text{N }}(0, \, \sigma_{\text{e}} )} \hfill \\ {{\text{Level}} - 2: \, \pi_{0} {\text{i }} = \beta_{0} {\text{i}} + {\text{u}}_{0} {\text{i}}\quad {\text{where}},{\text{ u}}_{\text{ti}} \sim {\text{N }}(0, \, \sigma_{\text{u}} )} \hfill \\ \end{array}$$

Where, the Greek letters π and β indicate first and second level parameter respectively.

By substituting, we get

2 $${\text{Y}}_{\text{ti}} = \beta_{00} + {\text{ u}}_{0} {\text{i}} + {\text{e}}_{0} {\text{i}}$$

π_0_i is the intercept for individual i for each response; β_00_is the mean intercept over all individuals, and u_0_i are the deviation of individual-specific residual; finally, e_0_i is the time-specific residual. Now, proportion variance or the intra class correlation (ICC) refers to a set of coefficients representing the relationship between variables of the same individuals decomposes into two independent components (i.e., level-1 and leve-2). Thus, ICC explained by the individuals (level-2) in the population is given by

3 $${\text{ICCCD}}4 = \frac{{\upsigma_{{{\text{u}}02}} }}{{\upsigma_{{{\text{u}}02}} +\upsigma_{{{\text{e}}02}} }}$$

Where, e_0_ and u_0_ for each response are different and ICCmeasures = 1 − ICCindividual.

### Random intercept model

A random intercepts model is a model in which intercepts are allowed to vary, and therefore, the scores on the dependent variable for each repeated measurement are predicted by the intercept that varies across patients. The prior models are sometimes called unconditional (intercept only) model; because there are no measured covariates used to predict the random effect.

Now, based on [10], the model often interested in assessing how a longitudinal outcome is associated with a covariate whose value changes over time such covariant are called time-varying covariates X_pi_, and whose value not changes over time called time invariant predictor Z_si_.

4 $${\text{The model is given by}}:{\text{ Level 1}}:{\text{Yti }} = \pi 0{\text{i}} + \pi_{{ 1 {\text{i}}}} {{\text{X}}_{\text{pi}} }+ {\text{e}}_{{0{\text{i}}}}$$

Time invariant covariates Z inter the equation at the second level.

5 $$\begin{aligned} {\text{Level 2}}:\pi_{0} {\text{i }} & = \beta_{00} + \, \beta_{0 1} {\text{Z}}_{\text{si}} + {\text{ u}}_{0} {\text{i}} \\ \pi_{ 1} {\text{i}} & = \beta_{ 10} + \, \beta_{ 1 1} {\text{Z}}_{\text{si}} \\ \end{aligned}$$

By substituting, we get a uni-variate random intercept model:-

6 $${\text{Y}}_{\text{ti}} = \beta_{00} + \beta_{ 10} {\text{X}}_{\text{pi}} + \beta_{0 1} {\text{Z}}_{\text{si}} + \beta_{ 1 1} {{\text{Z}}_{\text{si}}} {\text{X}}_{\text{pi}} + {\text{u}}_{0} {\text{i }} + {\text{e}}_{0} {\text{i}}$$

Where, β_00_ is the overall average intercept for each response, β_10_ is the slop of time varying covariates, β_01_ is the slop of time invariant covariates, β_11_ is the mean difference change between time varying covariates and time invariant covariates, and lastly e_0_i and u_0_i are still the within and between individual error term of the intercept. Therefore, in this model β_00_ + β_10_X_pi_ + β_01_Z_si_ + β_11_Z_si_X_pi_ are the fixed part, because the coefficients are fixed.

The remaining u_0_i + e_0_i are called the random part of the model. Where, X_pi_, p = 1, 2,…, P denotes the P time varying covariates that were included in the analysis, and Z_si_, s = 1, 2… S denotes the s invariant covariates that were included in the analysis.

### Random coefficients model

This random coefficients model is a model in which slopes are allowed to vary in addition to intercepts for each uni-variate response. The relationship between an explanatory variable and the response is different across all patients with their intercept and slope by considering time varying covariate and time invariant covariate.

7 $${\text{The model is given by}}:{\text{ Level 1}}:{\text{ Y}}_{\text{ti}} = \pi_{0} {\text{i}} + \pi_{ 1} {\text{iX}}_{\text{pi}} + {\text{e}}_{0} {\text{i}}$$

Time invariant covariates Z inter the equation at the second level.

8 $$\begin{aligned} {\text{Level 2}}: \, \pi_{0} {\text{i}} & = \beta_{00} + \, \beta_{0 1} {\text{Z}}_{\text{si}} + {\text{ u}}_{0} {\text{i}} \\ \pi_{ 1} {\text{i }} & = \beta_{ 10} + \, \beta_{ 1 1} {\text{Z}}_{\text{si}} + {\text{ u}}_{ 1} {\text{i}} \\ \end{aligned}$$

By substituting, we get uni-variate random coefficient model:-

9 $${\text{Y}}_{\text{ti}} = \beta_{00} + \beta_{ 10} {\text{X}}_{\text{pi}} + \beta_{0 1} {\text{Z}}_{\text{si}} + \beta_{ 1 1}{ {\text{Z}}_{\text{si}}} {\text{X}}_{\text{pi}} +{ {\text{u}}_{ 1}} {\text{iX}}_{\text{pi}} + {{\text{u}}_{0}} {\text{i}} + {\text{e}}_{0} {\text{i}}$$

Where, the only additional term in this model is u_1_i the random slope of time varying covariates for response.

### Selection of covariance structure

The most common covariance structures in repeated measure are: First, Simple structure species that the observations are independent, even on the same patient, and have homogeneous variance. Second, Compound symmetric structures were the observations on the same patient has homogeneous covariance and homogeneous covariance. Third, unstructured structure species no patterns in the covariance matrix, and is more appropriate for balanced data nature. Fourth, Autoregressive (order 1) covariance structure species homogeneous variance; and more appropriate for unbalanced data and equally spaced measurement times such that t_n_ + 1 − t_n_ is a constant for all n [12]. In addition to this, AR (1) model used only two parameters that are considerable superior than totally unstructured model even though − 2RLL is larger or worse [13].

### Variable selection for multilevel analysis

In order to select variables to be included in multi-variable analysis, forward variable selection was used. The first step in this selection is to fit a univariate multilevel model for each covariate at the 25% level. Next univariate model is fitted that contains all covariates that are significant in univariable analysis.

### Model selection and comparison

In order to select the best and final model which is appropriately fits with the given longitudinal data, it is necessary to compare the different models by using different techniques and methods. Hence, Akaki information criteria (AIC) and Bayesian information criteria (BIC) that calculated from deviance based on number of estimated parameter p is also most convenient at 5% level of significance, but for multilevel model deviance information criteria (DIC) is appropriate. After all, Deviance compares Chi squared distribution with degrees of freedom equal to the difference (p) in the number of parameters fitted under the two models smaller values is better [10].

### Parameter estimation

Parameter estimates of multilevel model were derived for both fixed components and random components. To estimate this component, there is different of parameter estimation technique. Among that, maximum likelihood estimation is most commonly used estimation method in multilevel model [14].

### Goodness of fit test

Once a model has been developed through different techniques in estimating the model parameters, there were several mechanisms involved in assessing the appropriateness, adequacy and usefulness of the model. First *t* test statistic is commonly used to test significance of individual parameter regression coefficients for each independent variable. Second, the deviance based test, or likelihood ratio test is a general principle for testing fixed multi-parameter and for testing about the random part of the model in applications of a hierarchical linear model.

### Model diagnosis

For multilevel analysis making inference about the model depends on whether the data met the required assumptions or not. In this hierarchical regression models some graph and other techniques were used to assess peculiarities or the distinctive features of the model with regards to the data. Therefore the assumptions in this hierarchical regression models were linearity, normality, heteroscedasticity and residuals nature using usual manner.

## Results

There were a total of 1408 visits from 216 subjects; the numbers of visits per subject were minimum 3 and a maximum of 10 visits with an equally 6-month interval for all patients. The sample sizes at the six consecutive time points were 216, 216, 196, 167, 142, 125, 105, 93, 80 and 68. Therefore, the measurements were sharply increasing the degree of missing over time due to dropouts, missed clinic visits and transfers.

From socio-demographic and clinical related covariates results were shown in Table 2 below. Among a total of 216 respondents 131 (60.6%) were female and 98 (44.9%), 68 (31.5%), 35 (16.2%) and 16 (7.4%) of respondents were married, divorced, single and widowed marital status respectively. Besides, the functional status of patients 194 (89.8%), 20 (9.3%) and 1 (0.5%) were working, ambulatory and bedridden. Moreover, the adherence status of patients 202 (93.5%), 9 (4.2%) and 4 (1.9%) were good, fair and poor respectively. Likewise, the clinical stages of patients 179 (82.9%), 17 (7.9%), 16 (7.4%) and 2 (0.9%) were stage I, stage II, stage III and stage IV respectively. Regarding the functional status of patients 194 (89.8%), 20 (9.3%) and 1 (0.5) were working, ambulatory and bedridden respectively and TB status 192 (88.9%) were negative.

**Table 2:** Socio-demographic and clinical related covariates

Variables	Levels	Count (%)	Variables	Mean (SE)	SD
Gender	Male Female	85 (39.4) 131 (60.6)	Weight	55.48 (0.698)	10.21
Marital status	Single Married Divorcé Widowed	35 (16.2) 97 (44.9) 68 (31.5) 16 (7.4)	Hemoglobin level	18.25 (2.252)	33.028
Adherence status	Good Fair Poor	202 (93.5) 9 (4.2) 4 (1.9)	Age	35.09 (0.699)	10.27
WHO clinical stage	I II III IV	179 (82.9) 17 (7.9) 16 (7.4) 2 (0.9)	Baseline CD4	301.40 (11.737)	171.7
Functional status	Working Ambulatory Bedridden	194 (89.8) 20 (9.3) 1 (0.5)	CD4 count	353.72 (12.879)	189.28
TB screen	Positive Negative	24 (11.1) 192 (88.9)			

As can be seen from Table 2, the mean of baseline CD4 for patients was 301.40 (SD = 171.7). Likewise; the overall mean of CD4 count for respondents was 353.72 (SD = 189.28). Furthermore, the mean with a standard deviation of weight, hemoglobin level, and age of patients was 55.48 (10.21), 18.25 (33.028) and 35.09 (10.27) respectively.

Table 3 shows the increment of mean value of CD4 count of patients who visit for ten consecutive. However, the standard deviation was slight decreased among the ten consecutive visited.

**Table 3:** Summary statistics of response variable at each follow-up

Number of visiting times	CD4 count	
	Mean	Standard deviation
1	353	189
2	366	208
3	381	220
4	398	208
5	387	219
6	419	195
7	434	196
8	449	189
9	450	179
10	482	189

### Individual profile plot of growth curve analysis

**Fig. 1:**
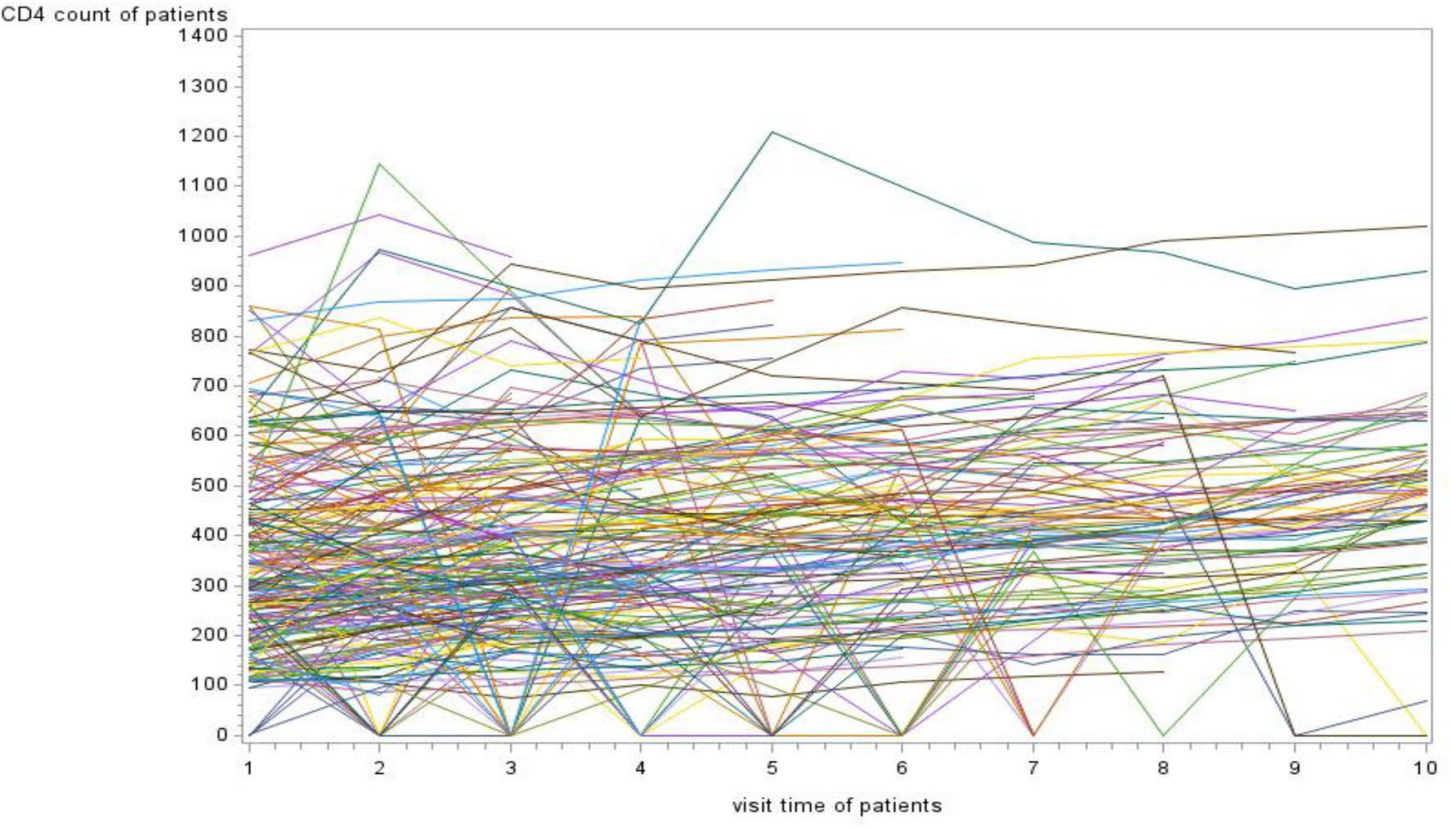
Individual profile plot of patients

The visualized the pattern of CD4 count measurements of the patient’s overtime and the overall individual plots were considered. Figure 1 indicated that the variability within and between patients was slightly decreasing trend on each respondent throughout the follow-up. For responses, most (but not all) observations were slightly turned down throughout the follow-up. Likewise, the variation within-subject throughout the time decreased each response from a visit to a visit. From Table 4 results show intra-class correlation gives strong evidence that variability was occurring between the patients. Therefore, the intra-class correlation of this study was 9923.8/9923.8 + 5843.87 = 0.6294. Therefore, 63% of the variation for CD4 count existed between patients and the remaining 37% of variation existing within patients.

**Table 4:** Covariance parameter estimates for CD4 count

Cov Parm	Subject	Estimate	P value
Variance	id	9923.80	0.0018
AR (1)	id	0.03525	0.001
Residual		5843.87	0.0201

Table 5 shows the random coefficient time-varying covariate model was well fitted because generalized Chi square/df was closed to one. Under this study Shapiro–Wilk test of normality was used and it was show normality with a P-value equal to 0.0001.

**Table 5:** Comparison of multilevel models analysis

Random effect	Generalized Chi Square/DF
Intercept only model	20.79
Random intercept time varying covariate model	30.19
Random intercept time invariant covariate model	50.05
Random coefficient time varying covariate model	8.49
Random coefficient time invariant covariate model	13.50

**Fig. 2:**
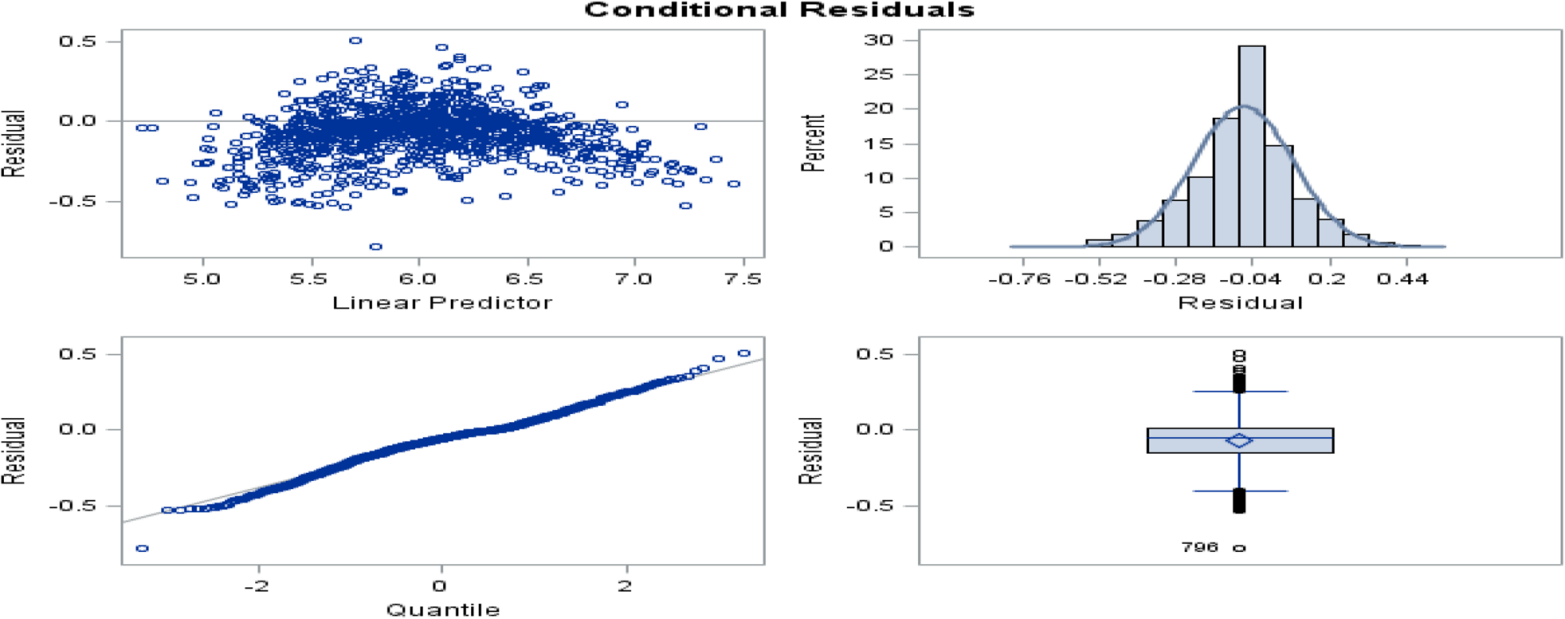
Residual and QQ plot

Besides residuals plot shown in Fig. 2, indicate that the residuals and the fitted values confirm linearity without distinct patterns and show constant variance and the QQ plot confirms normality of errors which was residual points follow the straight dashed line. Therefore, the overall assumptions satisfied and fit the random coefficient time-varying covariate model with the selected covariates show in Table 5.

From the fitted univariate multilevel model was modeled with sets of covariates that include fixed effect parameters age, marital status, weight, hemoglobin level, functional status, WHO stage, TB screen, opportunistic infection, and ART adherence. Among those covariates, there was significance difference by intercept, weight and hemoglobin level for a CD4 count of patients. According to results intercepts, weight and hemoglobin levels were positively associated with a CD4 count of patients. Therefore, this study shows that CD4 cell count of patients increased as weight and hemoglobin levels increase (Table 6).

**Table 6:** Parameter estimation of random coefficient time varying covariate model

Effect	levels	Estimate	Standard error	Pr > |t|	Lower	Upper
Intercept		4.5873	0.2891	0.0039	3.3434	5.8311
Age	Continuous	0.001465	0.002759	0.5955	− 0.00395	0.006882
Marital status	Single	− 1.2713	0.4372	0.1007	− 3.1524	0.6099
Marital status	Married	− 0.2020	0.2306	0.4734	− 1.1940	0.7900
Marital status	Divorced,	− 0.2088	0.2205	0.4436	− 1.1577	0.7400
Marital status	Widowed	0				
Weight	Continuous	0.009483	0.000434	<.0001	0.008631	0.01033
Homoglovenlevel	Continuous	0.06317	0.001454	<.0001	0.06032	0.06603
Functional status	Working,	0.07050	0.07957	0.3791	− 0.08866	0.2297
Functional status	Ambulatory	0.05850	0.08048	0.4702	− 0.1025	0.2195
Functional status	Bedridden	0	.	.	.	.
WHO stage	I	− 0.09848	0.07262	0.1782	− 0.2426	0.04565
WHO stage	II	− 0.1129	0.07629	0.1421	− 0.2643	0.03850
WHO stage	III	− 0.09694	0.07721	0.2123	− 0.2502	0.05631
WHO stage	IV	0	.	.	.	.
TB screen	Positive	− 0.03879	0.02496	0.1249	− 0.08861	0.01104
TB screen	Negative	0	.	.	.	.
Opportunistic infection	No	0.07982	0.04005	0.0594	− 0.00347	0.1631
Opportunistic infection	Yes	0	.	.	.	.
ART adherence	Good	0.09495	0.1032	0.3712	− 0.1238	0.3137
ART adherence	Fair	0.07064	0.1104	0.5315	− 0.1635	0.3048
ART adherence	Poor	0	.	.	.	.

## Discussion

Highly active antiretroviral therapy has greatly reduced morbidity and mortality in patients with human immunodeficiency virus. The effective antiretroviral therapy was important intervention in terms of improving longevity and preventing opportunistic infections in patients with human immunodeficiency virus infection. The literature and this study shows antiretroviral therapy is effective treatment for human immunodeficiency virus to reduce the viral load to undetectable levels. The studied patients received the same type of antiretroviral due to the therapeutic effectiveness and adherence. Antiretroviral drug were associated with weight gain and hemoglobin level. Human immunodeficiency virus has damaged immune system and reduced CD4 count. Highly active antiretroviral therapy will strength immune system, extend life and increased CD4 count. Weight and hemoglobin level increased due to patients received antiretroviral therapy.

Based on different well-organized literature, some discussions were organized as follows:-

In this study, the random coefficient time-varying covariate model was well fitted because generalized Chi square/df was 8.49 which closed to one. From the multilevel analysis, more than half percent of the variation was explained from between patients; and the remaining is explained by within patients. The finding is consistent with [9]  that for any repeated data between variations are higher than within variation. According to this study, weight was an important predictor for a CD4 count of patients that as weight increase in 1 kg then the mean log of CD4 will be increased by 0.009483 cells per milliliter of blood (Table 6). This estimated result also consistent with similar previous studies conducted by different scholars [15, 16]. Lastly, this finding provides that the log of CD4 count of patients will be increased by 0.06317 when the hemoglobin level of patients increased by one (Table 6). This result consistent with a previous finding [17].

## Conclusion

This study used a series of repeated measurements over time at the lowest level is nested with the individual patients at the highest level. Such nested structures are typically strong hierarchies because 63% of the variation for CD4 count existed between patients and the remaining 37% of variation existing within patients. Therefore, multilevel modeling considered variability between, and within patients. This study determined the determinants of CD4 cell count among antiretroviral therapy attendants of HIV infected adults follow up in Gonder teaching referral hospital, Gonder, Ethiopia.

From this study, hemoglobin level and weight of patients were statistically significant at a 5% level of significance for the log of CD4 count of patients follow up in Gonder teaching referral hospital, Gonder, Ethiopia. Moreover, the result of the study shows that the log of CD4 count of patients increased when hemoglobin level and weight of patients increased.

## Data Availability

The raw data used in this study can be accessed from the Gondar Teaching Referral Hospital.

## Abbreviations

SAS: Statistical analysis system
AIDS: Acquired immunodeficiency syndrome
HIV: Human immunodeficiency virus
WHO: World health organization
ART: Antiretroviral therapy
GC: Gregorian calendar
R^2^: Coefficient of determination
ICC: Intra class correlation
SD: Standard deviation
AIC: Akaki information criteria
BIC: Bayesian information criteria
DIC: Deviance information criteria
CD4: Cluster of differentiation four
HAART: Highly active antiretroviral therapy
TB: Tuberculosis
UNAIDS: United nation report on acquired immune deficiency syndrome
DF: Degree freedom
