# Correction to: Multilevel model on longitudinal data analysis in determinants of CD4 cell count among antiretroviral therapy attendant of HIV infected adults follow up in Gondar Teaching Referral Hospital, Gonder, Ethiopia

**DOI:** 10.1186/s12981-021-00373-9

**Published:** 2021-08-09

**Authors:** Kindu Kebede

**Affiliations:** Department of Statistics, College of Computing and Informatics, Haramaya University, Harar, Ethiopia

## Correction to: AIDS Res Ther (2021) 18:5 10.1186/s12981-020-00329-5

Following publication of the original article [1], the authors realised that in the Declarations section, under “Ethical approval and consent to participate”, Bahir Dar University was erroneously mentioned as the institution providing ethical clearance. The error was made in good faith due to a temporary change in affiliation of the corresponding author. However, the correct institution to quote for ethical clearance is Haramaya University. The correct ethics statement should be as follows:

Letter of ethical clearance was obtained from Haramaya University, Department of Statistics and submitted to the University of Gonder Teaching Referral Hospital to get permission to conduct the research. This study was developed in accordance with established legislation and complies with the norms of good clinical practice, and informed consent was being not necessary as personal identifying information was kept separate from the research data. Finally, the study protocol was approved by the ethics committee or medical directors of the University of Gonder Teaching Referral Hospital.

The authors would like to apologise for this mistake and are very sorry for the inconvenience caused.
